# Supplementary material for: Network Analysis of Anxiety Symptoms in Front-Line Medical Staff during the COVID-19 Pandemic
Source: Brain Sci. 2023 Aug 1;13(8):1155. doi: 10.3390/brainsci13081155 (PMC10452648; doi:10.3390/brainsci13081155)
Supplement: Supplementary file 1 [file brainsci-13-01155-s001.zip › brainsci-2487813-supplementary.pdf]

Supplementary Materials

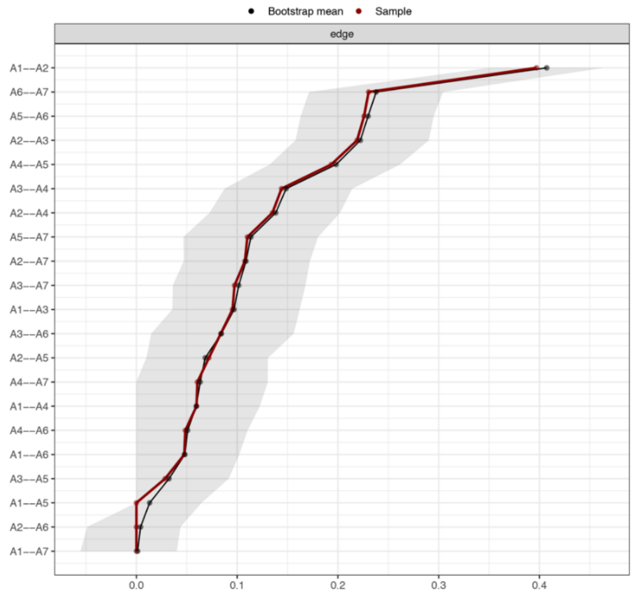

Figure S1 The accuracy of edge weighs between GAD-7 anxiety symptoms

Note: The red line represents the edge weight of the study sample. The black line represents the average edge weight evaluated by the bootstrap method. The grey area represents the confidence interval obtained by bootstrapping.

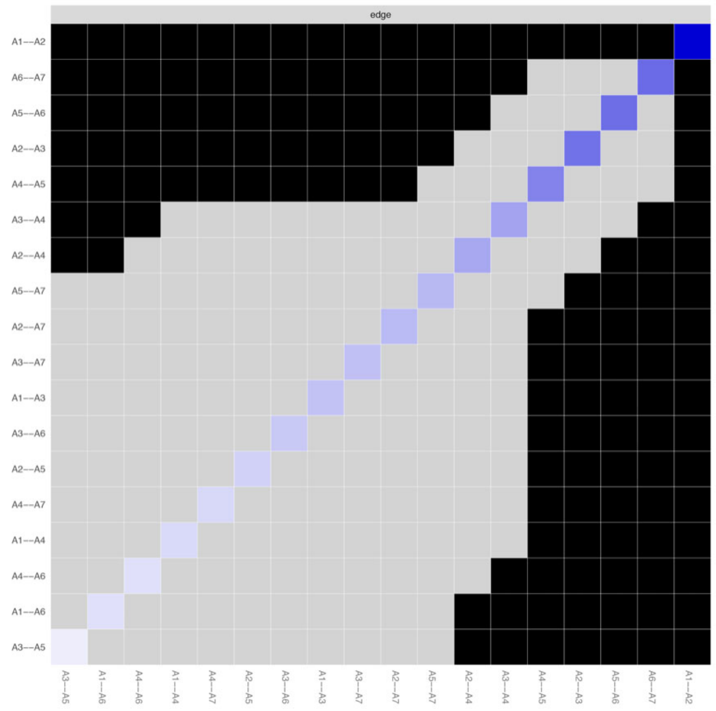

Figure S2 The difference test of edge weights between GAD-7 anxiety symptoms

Note: The gray squares represent that there is no statistical difference in the weights of the two corresponding edges. The black squares represent that the weights of the two corresponding edges are statistically different. The squares on the diagonal represent the color of edge weights in the variable network (Fig. 1). Blue represents positive relationship. Red represents negative relationship.

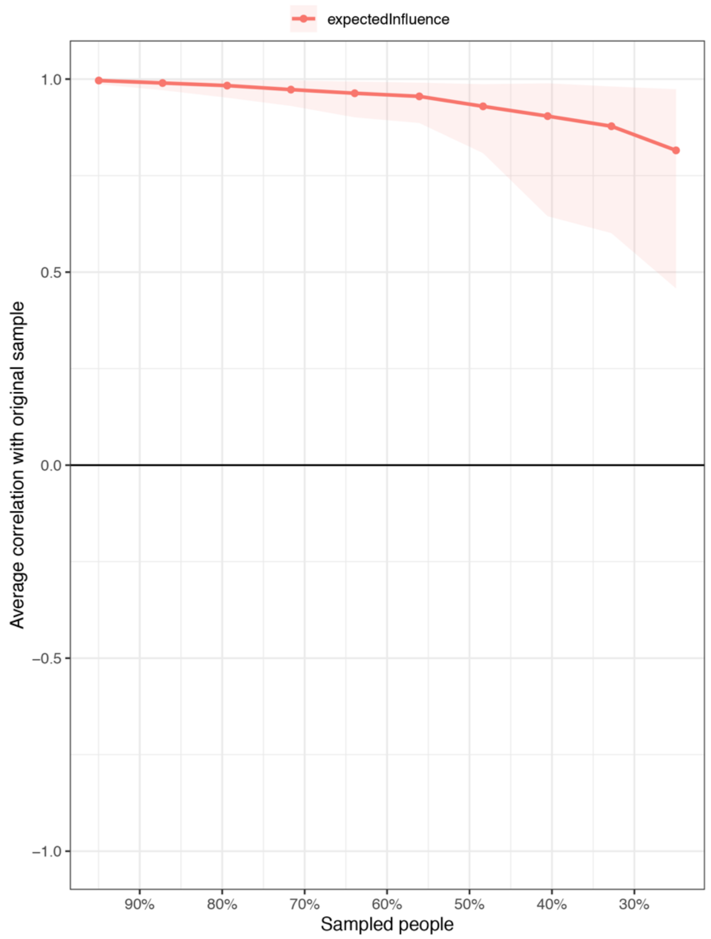

Figure S3　The CS coefficient of GAD-7 anxiety symptoms' EI

Note: The red line represents the average correlation between the EI of the original sample and the sub sample. The red area indicates the range from 2.5 to 97.5 quantiles.

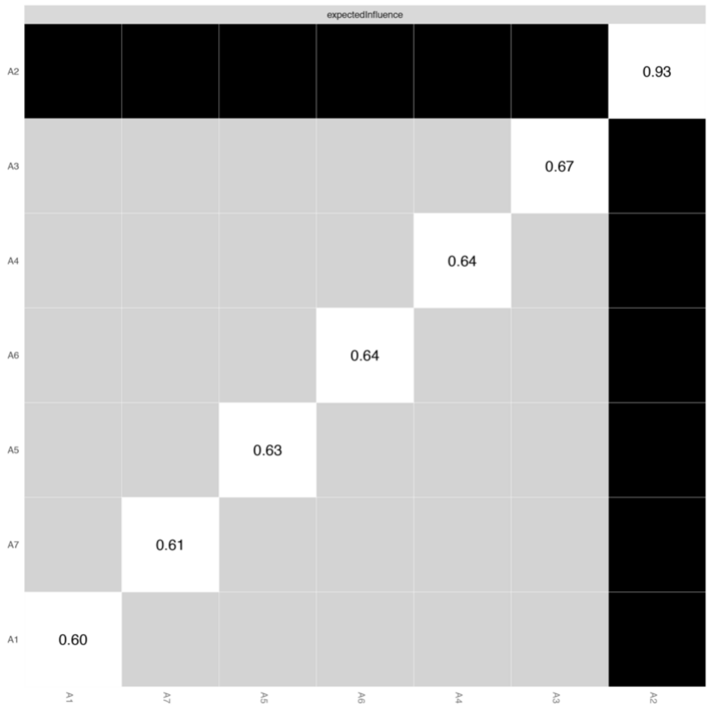

Figure S4 The difference test of GAD-7 anxiety symptoms' EI

Note: The grey squares represent that there is no statistical difference between the EI of the two corresponding nodes. The black squares indicate that the EI of the two corresponding nodes is statistically different. The numbers in the white square on the diagonal represent the EI value (row score) of the node.
